# MRI-Based Circumferential Strain in Boys with Early Duchenne Muscular Dystrophy Cardiomyopathy

**DOI:** 10.3390/diagnostics14232673

**Published:** 2024-11-27

**Authors:** Zhan-Qiu Liu, Nyasha G. Maforo, Patrick Magrath, Ashley Prosper, Pierangelo Renella, Nancy Halnon, Holden H. Wu, Daniel B. Ennis

**Affiliations:** 1 Department of Radiology, Stanford University, Palo Alto, CA 94305, USA; 2 Cardiovascular Institute, Stanford University, Palo Alto, CA 94305, USA; 3 Physics and Biology in Medicine Interdepartmental Program, University of California, Los Angeles, CA 90095, USA; nyasha.maforo@pennmedicine.upenn.edu (N.G.M.); holdenwu@mednet.ucla.edu (H.H.W.); 4 Department of Radiological Sciences, University of California, Los Angeles, CA 90095, USA; lafeir.lew@gmail.com (P.M.); aprosper@mednet.ucla.edu (A.P.); prenella@choc.org (P.R.); 5 Department of Bioengineering, University of California, Los Angeles, CA 90095, USA; 6 Department of Medicine, Division of Pediatric Cardiology, CHOC Children’s Hospital, Orange, CA 92868, USA; 7 Department of Pediatrics, University of California, Los Angeles, CA 90095, USA; nhalnon@mednet.ucla.edu; 8 Maternal & Child Health Research Institute, Stanford University, Palo Alto, CA 94305, USA

**Keywords:** Duchenne muscular dystrophy, cardiomyopathy, cardiovascular magnetic resonance imaging, late gadolinium enhancement, strain imaging, MRI tagging, left ventricular ejection fraction

## Abstract

**Background:** In boys with Duchenne muscular dystrophy (DMD), cardiomyopathy has become the primary cause of death. Although both positive late gadolinium enhancement (LGE) and reduced left ventricular ejection fraction (LVEF) are late findings in a DMD cohort, LV end-systolic circumferential strain at middle wall (E_cc_) serves as a biomarker for detecting early impairment in cardiac function associated with DMD. However, E_cc_ derived from cine Displacement Encoding with Stimulated Echoes (DENSE) has not been quantified in boys with DMD. We aim to: (1) use cine DENSE to quantify regional E_cc_ in LGE negative (-) boys with DMD and healthy controls; and (2) compare E_cc_ with LVEF in terms of differentiating DMD boys with LGE (-) from healthy boys. **Methods:** 10 LGE (-) boys with DMD and 12 healthy boys were enrolled prospectively in an IRB-approved study for CMR at 3T. Navigator-gated cine DENSE was used to obtain short-axis mid-ventricular data and estimate global and regional E_cc_. Group-wise differences were tested via a Wilcoxon rank-sum test. Within-group differences were tested via a Skillings-Mack test followed by pairwise Wilcoxon signed-rank tests. A binomial logistic regression model was adopted to differentiate between DMD boys with LGE (-) and healthy boys. **Results:** When compared to healthy boys, LGE (-) boys with DMD demonstrated significantly impaired septal E_cc_ [−0.13 (0.01) vs. −0.16 (0.03), *p* = 0.019]. In comparison to the E_cc_ in other segments, both groups of boys exhibited significantly reduced septal E_cc_ and significantly elevated lateral E_cc_. Septal E_cc_ outperformed LVEF in distinguishing DMD boys with LGE (-) from healthy boys. **Conclusions:** Reduced septal E_cc_ may serve as an early indicator of cardiac involvement in LGE (-) DMD boys prior to reduced LVEF and a positive LGE finding.

## 1. Introduction

Duchenne muscular dystrophy (DMD) is a life-threatening hereditary disease, occurring in approximately 2.63 to 11.66 out of every 10,000 male births [1]. DMD results in progressive skeletal, respiratory, and cardiomyopathy challenges, eventually leading to loss of respiratory function and ambulation, as well as heart failure [2]. Due to the advancements in respiratory clinical management, cardiomyopathy has emerged as the primary cause of mortality in DMD [3].

Cardiac magnetic resonance imaging (CMR) exams have proven useful for evaluating cardiac involvement in the later stages of DMD. Late gadolinium enhancement (LGE) is the current gold standard CMR technique that helps identify focal myocardial fibrosis. Positive LGE findings are associated with systolic dysfunction [4]. However, positive LGE findings are a mid- to late-stage finding in DMD [5,6], with an average onset at 15.2 ± 5.1 years [4] and highly variable associated outcomes [7,8]. Identifying the onset and progression of cardiac involvement for a specific boy is becoming more important as therapeutic options are evaluated and become increasingly available. Additionally, LGE MRI requires contrast administration, which has modest acceptance by pediatric subjects and their families and adverse effects, while rare, should be considered. Accordingly, there is an increasing interest to find non-contrast CMR biomarkers to assess early cardiac engagement in DMD boys prior to the appearance of LGE.

As a non-contrast biomarker, declined left ventricular ejection fraction (LVEF < 45%) serves as a significant predictor of fatal and nonfatal cardiovascular outcomes [9]. However, the onset of a measurable decline in LVEF is also a late outcome with an average onset at 16.2 ± 4.8 years [10]. Decreased EF also has a variable onset during the lifetime of a specific DMD patient. For example, LVEF was relatively preserved (LVEF > 45%) in many pediatric subjects with DMD and the presence of positive transmural LGE findings [11]. Because the onset and progression of DMD cardiomyopathy is subtle and highly variable [7], we aim to identify a sensitive non-contrast biomarker for assessing cardiac engagement in DMD boys prior to the impairment in LVEF or a positive LGE finding.

Alternatively, other emerging non-contrast CMR biomarkers have shown promise in identifying and tracking the progression of cardiomyopathy in DMD [12]. Reduced (less negative) peak mid-wall circumferential strain (E_cc_) derived from CMR tagging has also been identified as an early non-contrast biomarker able to distinguish between DMD patients and normal controls before the occurrence of reduced LVEF or a positive LGE finding [12,13,14]. Additionally, mid-ventricular E_cc_ was reported to be a sensitive biomarker of cardiac dysfunction prior to reduced LVEF or a positive LGE finding [15,16,17]. Impaired myocardial contractility was indicated by decreased (less negative) circumferential strain early [14]. Alternatively, cine Displacement Encoding with Stimulated Echoes (DENSE) is a well-validated technique for quantifying left ventricular (LV) E_cc_ [18]. DENSE has proven to be sensitive to changes in E_cc_ in acute myocardial infarction (MI) and hypertrophic cardiomyopathy [19]. Additionally, DENSE outperforms tagging with noticeable imaging and post-processing advantages [20]. To date, however, no report is currently available for E_cc_ derived from cine DENSE in a DMD cohort. Thus, we aim to: (1) To characterize and compare global and regional LV E_cc_ between healthy boys and LGE negative (-) boys with DMD (without detectable focal myocardial fibrosis); and (2) To identify a binomial logistic regression model able to differentiate DMD boys with LGE (-) from healthy boys using LV E_cc_ and LVEF.

## 2. Methods

### 2.1. Study Enrollment

LGE (-) boys with DMD (*N* = 10, 12.5 ± 3.0 years) and sex-matched and age-matched healthy controls (*N* = 12, 13.0 ± 2.0 years) were prospectively enrolled in a multi-center study. The study was compliant with Health Insurance Portability and Accountability Act, (HIPAA) and approved by the University of California, Los Angeles Institutional Review Board (IRB #16-000297) between January 2017 and January 2020. Parental consent and informed consent (or assent) statements were obtained from each participant. Healthy controls and boys with DMD were recruited at one of two children’s hospitals via referral. The demographics of the two groups is summarized in Table 1.

### 2.2. CMR Imaging

All subjects participated a 3T CMR exam (Skyra, Siemens Healthineers, Erlangen, Germany) using identical software, coils, and imaging protocols.

*Cine Imaging.* Standard functional imaging was acquired using a free-breathing retrospectively binned balanced steady state free precession (bSSFP) cine sequence [21,22] (6/8 partial Fourier and parallel imaging with acceleration factor of 4, matrix = 192 × 144, spatial resolution = 1.9 × 1.9 mm^2^, temporal resolution = 64.4 ms, TE/TR = 1.2/2.4, flip angle = 40°, bandwidth = 930 Hz/Px, slice thickness = 8 mm).

*Cine DENSE Imaging.* Acquisitions of mid-ventricular LV short-axis slices were performed with a navigator-gated free-breathing cine DENSE sequence [20] (2-point phase cycling, spatial resolution = 2.5 × 2.5 × 8 mm^3^, TE/TR = 1.2/15, k_e_ = 0.08 cycles/mm, spirals = 10, number of averages = 3, scan time ~2.5 min). Free-breathing acquisitions are important in patients with current or anticipated respiratory dysfunction owing to the impact of respiratory dysfunction on ventricular mechanics compared to breath holding [23].

*LGE Imaging*. Patients were imaged post-contrast (0.1 mMol/kg gadobenate dimeglumine, MultiHance) using a free breathing motion corrected phase sensitive inversion recovery (PSIR) sequence [24] (parallel imaging with acceleration factor of 2, matrix = 192 × 120, spatial resolution = 1.4 × 1.4 mm^2^, temporal resolution = 35.1 ms, TE/TR = 2.01/2.83, flip angle = 20°, bandwidth = 800–1300 Hz/Px, slice thickness = 6 mm). Images were acquired with full LV short axis coverage, as well as the vertical and horizontal long axis (VLA, HLA) views.

### 2.3. Post-Processing and Analysis

*Cine and LGE Analysis.* Two expert clinicians (PR or AP, both >8 years of experience) calculated LVEF from bSSFP cine images using commercial segmentation software (Circle CVI42, Circle Cardiovascular Imaging Inc., Calgary, AB, Canada) or Medis (Medis Cardiovascular Imaging). In DMD, a normal LVEF was classified as LVEF ≥ 55%, while a mild LVEF was defined as an LVEF between 45% and 54% [10,25]. The clinicians assessed the LGE images for the absence of positive LGE findings to identify the LGE (-) boys with DMD from amongst a larger cohort of enrolled subjects. The experts then computed the following functional metrics: LV end systolic and end diastolic volume (LVESV, LVEDV), LVEF, LV mass (LVM), RV end systolic and end diastolic volume (RVESV, RVEDV), RVEF, and RV mass (LVM). Indexed measures (LVESVi, LVEDVi, LVMi, RVESVi, RVEDVi, and RVMi) were derived by dividing by the estimated body surface area (BSA).

**Figure 1:**
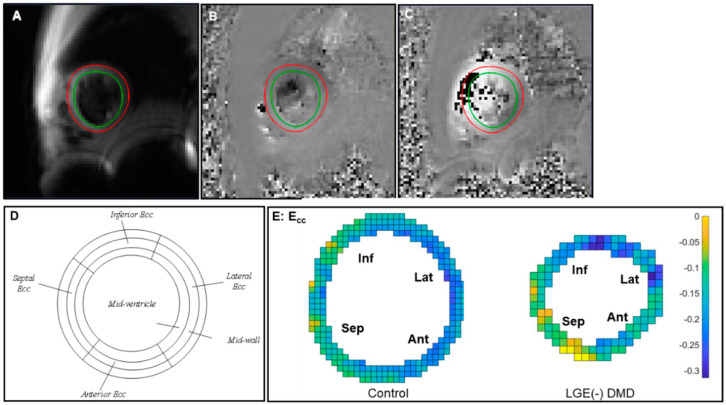
Representative end-systolic 2D cine DENSE CMR images and end-systolic strain maps from one mid-ventricular short-axis slice of left ventricle viewed from the apex. (**A**) A magnitude-reconstructed image; (**B**) a phase image encoded for x-displacement; (**C**) a phase image encoded for y-displacement; (**D**) diagram of mid-ventricular myocardial segmentation on LV free wall; (**E**) example of E_cc_ strain distribution in septum (sep), anterior wall (ant), lateral wall (lat), and inferior wall (inf).

*Cine DENSE Analysis.* LV borders were semi-automated segmented over the entire cardiac cycle (Figure 1A) via the open-source DENSEanalysis tool [26,27]. The strain analysis was proceeded with the pipeline described by Spottiswoode et al. [28]. In brief, after semi-automatic phase unwrapping, the 2D Lagrangian displacement field was estimated, spatially differentiated, and used to compute the regional strain tensor, thereby resulting in E_cc_ at each voxel. Subsequently, regional E_cc_ was averaged within four wall segments (septal, inferior, lateral, and anterior wall segments, Figure 1D). Reduced (less negative) E_cc_ indicates impaired end-systolic E_cc_ of the mid-ventricular LV myocardium.

### 2.4. Statistics

All statistical analyses were conducted in MATLAB (*p*-value < 0.05). All data are presented as median (IQR). A Wilcoxon rank-sum test was used to compare the demographics and LVEF for two cohorts, the LGE (-) boys with DMD and healthy controls. For each wall segment, a Wilcoxon rank-sum test was used to test group-wise differences in the LV regional E_cc_. For each group, a Skillings-Mack test followed by pairwise Wilcoxon signed-rank tests were used to test the regional differences in E_cc_ among the four wall segments.

*Binomial Logistic Regression*. A binomial logistic regression model tested whether global and regional E_cc_ and LVEF can distinguish between DMD boys with LGE (-) and healthy boys. Receiver operating characteristic (ROC) curves were used to present the results. The predictive capability of each biomarker was demonstrated by the area under the curve (AUC). Finally, a generalized linear regression model incorporating E_cc_ and LVEF was computed and compared to each biomarker individually using ROC analysis and AUC.

*Best Fitting Regression Model*. A framework for constructing a best fitting regression model was adopted to determine which predictors from LV and RV functional metrics had the greatest impact on predicting either global or regional E_cc_ that is significantly different in DMD boys with LGE (-) compared to healthy boys. Additional materials explain the framework in detail (Supplementary Material S1) and describe the mathematical formulations used in this analysis (Supplementary Material S2).

## 3. Results

### 3.1. Demographics

Compared to healthy boys, LGE (-) boys with DMD were significantly shorter [133 (18) cm vs. 165 (22) cm, *p* = 0.0007], resulting in significantly smaller BSA values [1.27 (0.49) m^2^ vs. 1.53 (0.37) m^2^, *p* = 0.032] (Table 1).

### 3.2. LV and RV Volume and Function

Four out of the 10 boys with DMD presented with mild LVEF (45–55%), but there was no significant difference in LVEF between the LGE (-) boys with DMD and healthy controls [58 (4) vs. 55 (10), *p* = 0.149]. 3 out of the 10 presented with mild RVEF (40–50% [29]). Of these, 2 had both mild LVEF and RVEF. There were no significant differences in the median of LVEF, LVEDVi, LVESVi, LVMi, RVEF, RVEDVi, RVESVi, or RVMi between the two groups, but the DMD boys with LGE (-) had significantly lower RVM, RVEDV, and RVESV compared to healthy boys (Table 2).

### 3.3. Global and Regional E_cc_

**Figure 2:**
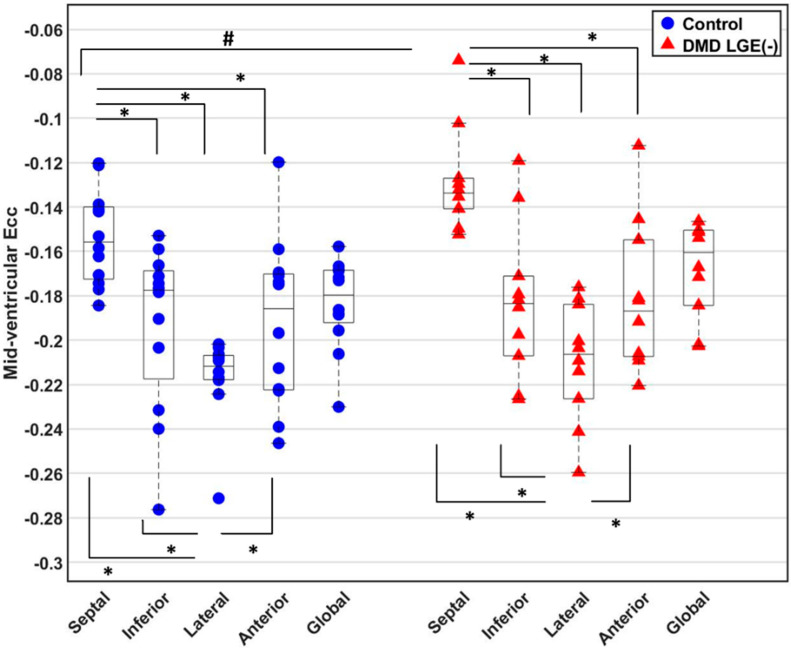
Comparison of E_cc_ between LGE (-) boys with DMD and healthy boys. For both the controls or LGE (-) DMD patients, septal E_cc_ was significantly reduced compared to inferior, lateral, and anterior E_cc_, while lateral E_cc_ was significantly elevated compared to septal, inferior, and anterior E_cc_. Additionally, the DMD patients with LGE (-) exhibited significantly impaired septal E_cc_ compared to healthy controls. * *p*-value ≤ 0.05 is significant for a comparison within either control or DMD boys with LGE (-) using Skillings-Mack test and then Wilcoxon signed-rank test for pairwise comparisons. # *p*-value ≤ 0.05 is significant for a comparison between DMD boys with LGE (-) and controls using Wilcoxon rank-sum test.

In Figure 1E, strain maps from representative subjects were used to display end-systolic E_cc_ at middle wall. Both healthy boys and DMD boys with LGE (-) exhibited regional differences in E_cc_. Compared with the E_cc_ in other circumferential segments, septal E_cc_ was impaired significantly and lateral E_cc_ was significantly higher (Figure 2). Septal E_cc_ was significantly impaired in LGE (-) boys with DMD compared to healthy boys [−0.13 (0.01) vs. −0.16 (0.03), *p* = 0.019] (Figure 2), but lateral wall E_cc_ was not significantly different between the LGE (-) boys with DMD and healthy controls. Additionally, there was no significant correlation between age and E_cc_ in the DMD cohort [Pearson’s correlation coefficient *R*^2^ = 0.036].

### 3.4. Binomial Logistic Regression

**Figure 3:**
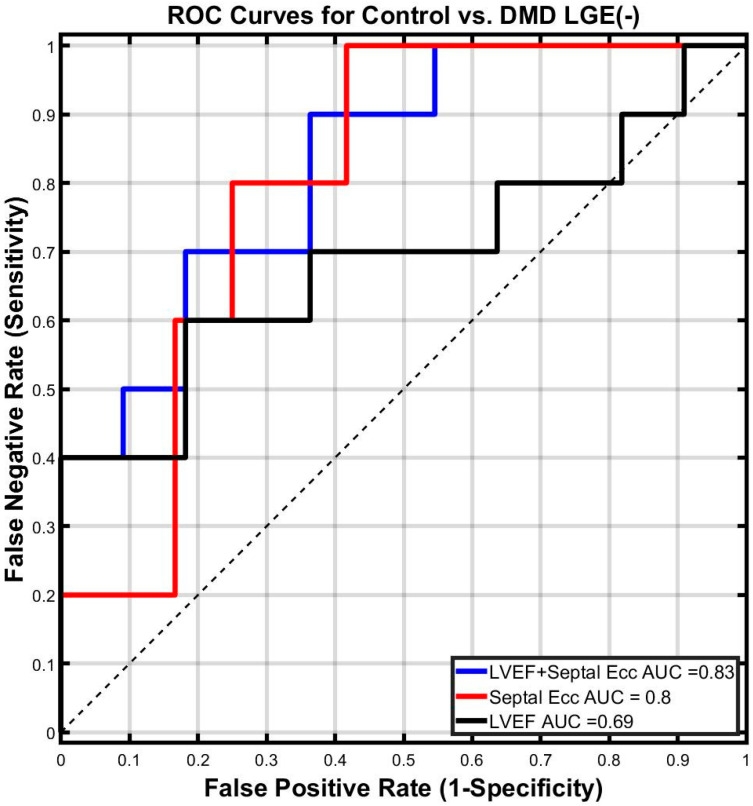
Receiver Operator Characteristic (ROC) curves for septal E_cc_ and LVEF derived from a binomial logistic regression model to differentiate LGE (-) boys with DMD from healthy boys. Larger area under the curve (AUC) values implies better performance in classification. Septal E_cc_ has the largest AUC among all individual biomarkers in differentiating DMD boys with LGE (-) from healthy boys. The combined logistical regression model of septal E_cc_ and LVEF outperforms each individual biomarker in distinguishing DMD boys with LGE (-) from healthy boys.

The AUC for septal E_cc_ was much larger than that of LVEF (AUC = 0.80 vs. AUC = 0.69). The use of septal E_cc_ in combination with LVEF improves the predictive capability of LVEF alone to distinguish between DMD boys with LGE (-) and healthy boys (AUC = 0.83 for LVEF combined with septal E_cc_ and 0.69 for LVEF) (Figure 3).

### 3.5. Best Fit Regression Model

Figure 2 indicates that Septal E_cc_ was significantly decreased in LGE (-) boys with DMD compared to healthy boys. A framework for determining the best fitting regression model was used to identify all predictors that are significantly correlated to the septal E_cc_ that are also significantly different between the two cohorts. As shown in Step 1 of Table 3, LVEF, LVEDV, and RVEDV were found to have significant predictor-by-group effects in predicting the pooled septal E_cc_. Subsequently, none of them were found to be highly correlated (*R*^2^ > 0.7) to the others (Step 2 of Table 3). Lastly, the best fitting regression model for predicting the septal E_cc_ in two cohorts:

*Pooled Septal E_cc_ ~ Group + LVEF + LVEDV + RVEDV + LVEDV× RVEDV + LVEF× Group* (1)

where the *p*-values are *p* = 0.013 for Group, *p* = 0.008 for LVEF, *p* = 0.011 for LVEDV, *p* = 0.010 for RVEDV, *p* = 0.010 for LVEF × Group, and *p* = 0.009 for LVEDV × RVEDV.

## 4. Discussion

As per our knowledge, this is the first report of reporting E_cc_ using free-breathing cine DENSE in LGE (-) boys with DMD with comparison to age-matched healthy boys. This is also the first report of a multi-variable binomial logistic regression model using only biomarkers not requiring an exogenous contrast agent to be implemented and tested in boys with DMD.

In our study, the DMD patients with normal LVEF and negative LGE were 11.8 ± 2.2 years old, comparable to the age of a similar cohort in the study by Hor et al. (13.6 ± 3.3 years old [16]). The DMD patients with mild LVEF and negative LGE were 13.3 ± 1.7 years old, which falls between the ages of similar patient groups in the study by Hor et al. (15.3 ± 4.4 years old [16]) and the study by Ashford et al. (10.6 ± 3.01 years old [14]). Septal E_cc_ was significantly lower (less negative) and lateral E_cc_ was significantly higher (more negative) both in LGE (-) boys with DMD and healthy controls. Previously, comparable results were reported using tagging in DMD patients and healthy controls [14,16]. The regional heterogeneity in E_cc_ is likely caused by regional differences in loading conditions (e.g., the septum is loaded by both RV and LV pressures, whereas the LV lateral wall is loaded by the LV pressure alone), tethering effects of the RV, ventricular interdependence [30], and microstructural differences between the septal and lateral wall [31,32,33]. In addition, septal E_cc_ in DMD boys with LGE (-) was significantly impaired compared to that of healthy boys. The best fitting regression model revealed that decreased LVEF, LVEDV, and RVEDV predicted the significantly reduced septal E_cc_ in LGE (-) boys with DMD compared to controls. This may be due to any of several reasons. First, the lower end diastolic volumes may not have optimal sarcomere length for contraction (Frank–Starling mechanism [34]), which could lead to impaired myocardial contractility as indicated by reduced E_cc_. Second, reduced septal E_cc_ was significantly correlated to decreased biventricular end diastolic volumes prior to the presence of focal fibrosis (LGE (+)), which may contribute to the fact that septum is the physical interface between two ventricles, where the mechanics of the two ventricles interact and the ventricles can affect each other. The septum was also previously reported as a highly ventricular interdependent functional unit [35]. However, the LV free wall E_cc_ was not significantly correlated to decreased biventricular end diastolic volumes because it may compensate similar to patients with septal infarction [36]. Lastly, the predictive effect of the best fitting regression model suggests that the reduced septal shortening may be contributed to abnormal biventricular end-diastolic loading conditions. Considering that the LV and RV are connected to the pulmonary circuit and the pulmonary circuit may not be normal in these patients, the changes in end-diastolic loading conditions may be attributed to chronic pulmonary insufficiency [37,38]. A previous study showed that respiratory system dysfunction resulting from DMD may also impact the biventricular end-diastolic loading conditions [37]. The decreased septal E_cc_ was likely attributed to the preceding respiratory system dysfunction, which may exacerbate the progression of cardiomyopathy in DMD patients. Future study should take the correlation of septal E_cc_ and pulmonary function data into account. Additionally, the effect of respiratory mechanics on the right ventricle and the septal function needs further study.

For the classification task of differentiating DMD boys with LGE (-) from healthy controls, septal E_cc_ outperformed LVEF. Additionally, the combination of septal E_cc_ and LVEF outperformed septal E_cc_ alone or LVEF alone.

Importantly, septal E_cc_ could be used as an earlier biomarker than LGE and LVEF for indicating the subtle beginning of LV cardiac engagement in DMD without the need for an exogenous contrast agent. Thus, this may enable more frequent, earlier, and better patient-specific treatment decisions.

*Limitations*. First, this multi-center study is limited by its sample size due to the challenges in recruiting boys with a complex and rare genetic disease as well as well-matched healthy controls. Nevertheless, the statistical methods provide significant findings. The best fit regression model necessarily overfitted septal E_cc_ due to the procedures of multiple testing and refitting. This may lead to optimistic *p*-values. Further evaluation of the regression model is needed. Finally, this study is limited by single-time-point evaluation of the subjects. Future work will include the evaluation of within-subject longitudinal changes in E_cc_ assessment to characterize the progression of cardiac involvement and to determine if E_cc_ could be longitudinal imaging end-points for clinical trials.

## 5. Conclusions

Using free-breathing cine DENSE, we showed septal E_cc_ was significantly decreased in DMD boys with LGE (-) compared with healthy boys. Declines in septal E_cc_ were significantly correlated to changes in LVEF, LVEDV, and RVEDV. Additionally, a binomial logistic regression model that combined septal E_cc_ and LV ejection fraction sensitively distinguished (AUC = 0.83) DMD boys with LGE (-) from healthy controls absent the need for an exogenous contrast agent. Thus, reduced septal E_cc_ may serve as an early non-contrast biomarker indicating the subtle beginning of cardiomyopathy in pediatric subjects with DMD prior to significantly reduced LVEF and a positive LGE finding.

## Figures and Tables

**Table 1:** Demographics of DMD boys with LGE (-) and healthy controls.

	Controls *N* = 12	DMD *N* = 10
Age (years)	13 (4.0) range (9–21)	12.5 (6.0) range (9–21)
Male (%)	100%	100%
Height * (cm)	165 (22)	133 (18)
Weight (kg)	51 (18)	46 (28)
BMI (kg/m^2^)	18.7 (6.7)	25.7 (12.6)
BSA * (m^2^)	1.53 (0.37)	1.27 (0.49)
Heart Rate (bpm)	78 (30)	93 (23)
Ambulatory (%)	12 (100%)	3 (30%)
Ventilatory Support (%)	0%	0%

Data is reported as median and interquartile range (IQR); HR, heart rate; BMI, body mass index; BSA, body surface area. * *p* < 0.05 compared to controls.

**Table 2:** Summary of LV and RV volume and function, as well as differences between healthy controls and DMD boys with LGE (-).

	Control *N* = 12	DMD LGE (-) *N* = 10	*p*-Value		Control *N* = 12	DMD LGE (-) *N* = 10	*p*-Value
LVEF (%)	58 (4)	55 (10)	0.149	RVEF (%)	54 (8)	54 (9)	0.921
LVEDVi (mL/m^2^)	84 (17)	87 (25)	0.972	RVEDVi (mL/m^2^)	83 (22)	81 (36)	0.249
LVESVi (mL/m^2^)	36 (5)	40 (15)	0.699	RVESVi (mL/m^2^)	39 (11)	34 (20)	0.223
LVMi (g/m^2^)	38 (14)	32 (12)	0.062	RVMi (g/m^2^)	31 (7)	25 (10)	0.199
LVEDV (mL)	141 (64)	93 (33)	0.057	RVEDV (mL)	142 (53)	87 (32)	0.004 *
LVESV (mL)	59 (25)	42 (17)	0.149	RVESV (mL)	61 (18)	37 (18)	0.004 *
LVM (g)	57 (45)	39 (12)	0.008	RVM (g)	49 (17)	31 (13)	0.006 *

* *p*-value ≤ 0.05 is significant.

**Table 3:** Step-wise results for the framework of discovering all biomarkers contribute to predicting the septal Ecc in DMD boys with LGE (-) and healthy controls.

All Available Predictors in the Study	Age, HR, Height, Weight, BSA, BMI, LVM, LVMi, LVESV, LVEDV, LVEF, LVESVi, LVEDVi, RVM, RVMi, RVESV, RVEDV, RVEF, RVESVi, RVEDVi	
**Step 1: Exclude derivable predictors**		
Remaining Predictors	Age, HR, Height, Weight, BSA^−1^, BMI, LVM, LVEDV, RVM, LVEF, RVEDV, RVEF	
**Step 2: Calculate predictor-by-group effect for each predictor**		
Regression Model	*Pooled Septal Ecc ~ constant + group + X + group × X*	
**Predictors (*X*)**	**Interaction Term (*group × X*)**	
	**Coefficients**	***p*** **-value**
LVEF	0.006	0.034 *
LVEDV	−0.00075	0.019 *
RVEDV	−0.00068	0.044 *
Remaining Predictors	LVEF, LVEDV, RVEDV	
**Step 3: Calculate inter-predictor correlations**		
**Predictors**		***R^2^***
LVEF & LVEDV		0.014
LVEF & RVEDV		0.021
LVEDV & RVEDV		0.500
Remaining Predictors		LVEF, LVEDV, RVEDV
**Step 4: Perform stepwise backward regression using the Akaike information criterion**		
Best Fitting Regression Model	*Pooled Septal Ecc ~ Group + LVEF + LVEDV + RVEDV + LVEDV×RVEDV + LVEF × Group*	
**Terms**	**Coefficients**	***p*** **-value**
(constant)	0.7329	0.012 *
Group	−0.3647	0.013 *
LVEF	−0.0130	0.008 *
LVEDV	−0.0015	0.011 *
RVEDV	−0.0011	0.010 *
LVEDV × RVEDV	0.00001	0.009 *
LVEF × Group	0.0067	0.010 *

* *p* < 0.05 is significant.

## Data Availability

The datasets used and/or analyzed during the current study are available from the corresponding author on reasonable request.

## Supplementary Materials

The following supporting information can be downloaded at: https://www.mdpi.com/article/10.3390/diagnostics14232673/s1, Supplementary Materials S1 and S2. References [39,40,41,42,43] are cited in the Supplementary Materials.

## References

[1] McNally E.M., Kaltman J.R., Benson D.W., Canter C.E., Cripe L.H., Duan D., Finder J.D., Groh W.J., Hoffman E.P., Judge D.P. (2015). Contemporary cardiac issues in Duchenne muscular dystrophy. Circulation.

[2] Bushby K., Finkel R., Birnkrant D.J., Case L.E., Clemens P.R., Cripe L., Kaul A., Kinnett K., McDonald C., Pandya S. (2010). Diagnosis and management of Duchenne muscular dystrophy, part 2: Implementation of multidisciplinary care. Lancet Neurol..

[3] Cheeran D., Khan S., Khera R., Bhatt A., Garg S., Grodin J.L., Morlend R., Araj F.G., Amin A.A., Thibodeau J.T. (2017). Predictors of Death in Adults With Duchenne Muscular Dystrophy–Associated Cardiomyopathy. J. Am. Heart Assoc..

[4] Hor K.N., Taylor M.D., Al-Khalidi H.R., Cripe L.H., Raman S.V., Jefferies J.L., O’Donnell R., Benson D.W., Mazur W. (2013). Prevalence and distribution of late gadolinium enhancement in a large population of patients with Duchenne muscular dystrophy: Effect of age and left ventricular systolic function. J. Cardiovasc. Magn. Reson..

[5] Tandon A., Villa C.R., Hor K.N., Jefferies J.L., Gao Z., Towbin J.A., Wong B.L., Mazur W., Fleck R.J., Sticka J.J. (2015). Myocardial fibrosis burden predicts left ventricular ejection fraction and is associated with age and steroid treatment duration in Duchenne muscular dystrophy. J. Am. Heart Assoc..

[6] Silva M.C., Meira Z.M.A., Giannetti J.G., da Silva M.M., Campos A.F.O., de Melo Barbosa M., Starling Filho G.M., de Aguiar Ferreira R., Zatz M., Rochitte C.E. (2007). Myocardial delayed enhancement by magnetic resonance imaging in patients with muscular dystrophy. J. Am. Coll. Cardiol..

[7] Bushby K., Finkel R., Birnkrant D.J., Case L.E., Clemens P.R., Cripe L., Kaul A., Kinnett K., McDonald C., Pandya S. (2010). Diagnosis and management of Duchenne muscular dystrophy, part 1: Diagnosis, and pharmacological and psychosocial management. Lancet Neurol..

[8] Mavrogeni S., Papavasiliou A., Giannakopoulou K., Markousis-Mavrogenis G., Pons M.R., Karanasios E., Nikas I., Papadopoulos G., Kolovou G., Chrousos G.P. (2017). Oedema-fibrosis in Duchenne Muscular Dystrophy: Role of cardiovascular magnetic resonance imaging. Eur. J. Clin. Investig..

[9] Solomon S.D., Anavekar N., Skali H., McMurray J.J.V., Swedberg K., Yusuf S., Granger C.B., Michelson E.L., Wang D., Pocock S. (2005). Influence of Ejection Fraction on Cardiovascular Outcomes in a Broad Spectrum of Heart Failure Patients. Circulation.

[10] Mehmood M., Hor K.N., Al-Khalidi H.R., Benson D.W., Jefferies J.L., Taylor M.D., Egnaczyk G.F., Raman S.V., Basu S.K., Cripe L.H. (2015). Comparison of right and left ventricular function and size in Duchenne muscular dystrophy. Eur. J. Radiol..

[11] Florian A., Ludwig A., Engelen M., Waltenberger J., Rösch S., Sechtem U., Yilmaz A. (2014). Left ventricular systolic function and the pattern of late-gadolinium-enhancement independently and additively predict adverse cardiac events in muscular dystrophy patients. J. Cardiovasc. Magn. Reson..

[12] Magrath P., Maforo N., Renella P., Nelson S.F., Halnon N., Ennis D.B. (2018). Cardiac MRI biomarkers for Duchenne muscular dystrophy. Biomark. Med..

[13] Hor K.N., Wansapura J., Markham L.W., Mazur W., Cripe L.H., Fleck R., Benson D.W., Gottliebson W.M. (2009). Circumferential strain analysis identifies strata of cardiomyopathy in Duchenne muscular dystrophy: A cardiac magnetic resonance tagging study. J. Am. Coll. Cardiol..

[14] Ashford M., Liu W., Lin S., Abraszewski P., Caruthers S., Connolly A., Yu X., Wickline S.A. (2005). Occult cardiac contractile dysfunction in dystrophin-deficient children revealed by cardiac magnetic resonance strain imaging. Circulation.

[15] Ryan T.D., Taylor M.D., Mazur W., Cripe L.H., Pratt J., King E.C., Lao K., Grenier M.A., Jefferies J.L., Benson D.W. (2013). Abnormal Circumferential Strain is Present in Young Duchenne Muscular Dystrophy Patients. Pediatr. Cardiol..

[16] Hor K.N., Kissoon N., Mazur W., Gupta R., Ittenbach R.F., Al-Khalidi H.R., Cripe L.H., Raman S.V., Puchalski M.D., Gottliebson W.M. (2015). Regional Circumferential Strain is a Biomarker for Disease Severity in Duchenne Muscular Dystrophy Heart Disease: A Cross-Sectional Study. Pediatr. Cardiol..

[17] Lang S.M., Shugh S., Mazur W., Sticka J.J., Rattan M.S., Jefferies J.L., Taylor M.D. (2015). Myocardial Fibrosis and Left Ventricular Dysfunction in Duchenne Muscular Dystrophy Carriers Using Cardiac Magnetic Resonance Imaging. Pediatr. Cardiol..

[18] Lin K., Meng L., Collins J.D., Chowdhary V., Markl M., Carr J.C. (2017). Reproducibility of cine displacement encoding with stimulated echoes (DENSE) in human subjects. Magn. Reson. Imaging.

[19] Wehner G.J., Suever J.D., Haggerty C.M., Jing L., Powell D.K., Hamlet S.M., Grabau J.D., Mojsejenko W.D., Zhong X., Epstein F.H. (2015). Validation of in vivo, 2D displacements from spiral cine DENSE at 3T. J. Cardiovasc. Magn. Reson..

[20] Zhong X., Spottiswoode B.S., Meyer C.H., Kramer C.M., Epstein F.H. (2010). Imaging three-dimensional myocardial mechanics using navigator-gated volumetric spiral cine DENSE MRI. Magn. Reson. Med..

[21] Kellman P., Chefd′hotel C., Lorenz C.H., Mancini C., Arai A.E., McVeigh E.R. (2009). High spatial and temporal resolution cardiac cine MRI from retrospective reconstruction of data acquired in real time using motion correction and resorting. Magn. Reson. Med..

[22] Xue H., Kellman P., LaRocca G., Arai A.E., Hansen M.S. (2013). High spatial and temporal resolution retrospective cine cardiovascular magnetic resonance from shortened free breathing real-time acquisitions. J. Cardiovasc. Magn. Reson..

[23] Reyhan M.L., Wang Z., Kim H.J., Halnon N.J., Finn J.P., Ennis D.B. (2017). Effect of free-breathing on left ventricular rotational mechanics in healthy subjects and patients with duchenne muscular dystrophy. Magn. Reson. Med..

[24] Kellman P., Larson A.C., Hsu L.-Y., Chung Y.-C., Simonetti O.P., McVeigh E.R., Arai A.E. (2005). Motion-corrected free-breathing delayed enhancement imaging of myocardial infarction. Magn. Reson. Med..

[25] Pfeffer M.A., Shah A.M., Borlaug B.A. (2019). Heart Failure with Preserved Ejection Fraction in Perspective. Circ. Res..

[26] Gilliam A., Scott A., vanMaanen D., Suever J. (2016). DENSEanalysis. https://github.com/denseanalysis/denseanalysis.

[27] Spottiswoode B.S., Zhong X., Lorenz C.H., Mayosi B.M., Meintjes E.M., Epstein F.H. (2009). Motion-guided segmentation for cine DENSE MRI. Med. Image Anal..

[28] Spottiswoode B.S., Zhong X., Hess A.T., Kramer C.M., Meintjes E.M., Mayosi B.M., Epstein F.H. (2007). Tracking myocardial motion from cine DENSE images using spatiotemporal phase unwrapping and temporal fitting. IEEE Trans. Med. Imaging.

[29] Kawel-Boehm N., Maceira A., Valsangiacomo-Buechel E.R., Vogel-Claussen J., Turkbey E.B., Williams R., Plein S., Tee M., Eng J., Bluemke D.A. (2015). Normal values for cardiovascular magnetic resonance in adults and children. J. Cardiovasc. Magn. Reson..

[30] Naeije R., Badagliacca R. (2017). The overloaded right heart and ventricular interdependence. Cardiovasc. Res..

[31] Kohl P. (2004). Cardiac cellular heterogeneity and remodelling. Cardiovasc. Res..

[32] Kvasnicka J., Vokrouhlický L. (1991). Heterogeneity of the myocardium. Function of the left and right ventricle under normal and pathological conditions. Physiol. Res..

[33] Solovyova O., Katsnelson L.B., Kohl P., Panfilov A.V., Tsaturyan A.K., Tsyvian P.B. (2016). Mechano-electric heterogeneity of the myocardium as a paradigm of its function. Prog. Biophys. Mol. Biol..

[34] Widmaier E., Raff H., Strang K. (2006). Cardiovascular patterns in health and disease. Vander’s Human Physiology: The Mechanism of Body Function.

[35] Saleh S., Liakopoulos O.J., Buckberg G.D. (2006). The septal motor of biventricular function. Eur. J. Cardiothorac. Surg..

[36] Asgeirsson D., Hedström E., Jögi J., Pahlm U., Steding-Ehrenborg K., Engblom H., Arheden H., Carlsson M. (2017). Longitudinal shortening remains the principal component of left ventricular pumping in patients with chronic myocardial infarction even when the absolute atrioventricular plane displacement is decreased. BMC Cardiovasc. Disord..

[37] Meyers T.A., Townsend D. (2015). Early right ventricular fibrosis and reduction in biventricular cardiac reserve in the dystrophin-deficient mdx heart. Am. J. Physiol. Heart Circ. Physiol..

[38] Larcher T., Lafoux A., Tesson L., Remy S., Thepenier V., François V., Le Guiner C., Goubin H., Dutilleul M., Guigand L. (2014). Characterization of Dystrophin Deficient Rats: A New Model for Duchenne Muscular Dystrophy. PLoS ONE.

[39] Moher D., Schulz K.F., Altman D., CONSORT Group (2001). The CONSORT Statement: Revised Recommendations for Improving the Quality of Reports of Parallel-Group Randomized Trials. JAMA.

[40] Harrell F.E. (2017). Regression modeling strategies. BIOS.

[41] Dobson A.J., Barnett A.G. (2018). An Introduction to Generalized Linear Models.

[42] McCullagh P. (2018). Generalized Linear Models.

[43] Akaike H. (1974). A new look at the statistical model identification. IEEE Trans. Autom. Control.

