# Supplementary material for: MRI-Based Circumferential Strain in Boys with Early Duchenne Muscular Dystrophy Cardiomyopathy
Source: Diagnostics (Basel). 2024 Nov 27;14(23):2673. doi: 10.3390/diagnostics14232673 (PMC11639811; doi:10.3390/diagnostics14232673)
Supplement: Supplementary file 1 [file diagnostics-14-02673-s001.zip › diagnostics-3281019-supplementary.pdf]

Supplementary Materials

S1

A framework for constructing a best fitting regression model was adopted in order to determine which predictors from LV and RV functional metrics had a significant impact on predicting either global or regional E_{cc} which is significantly different in DMD boys with LGE(-) compared to controls.

There were 20 predictors measured in the study. LVESV, LVEDV, and predictors indexed by BSA were excluded because they were products of other measured predictors (shown in the supplementary section for mathematical formulations of derivable predictors). This left 12 initial candidate predictors (Figure S1).

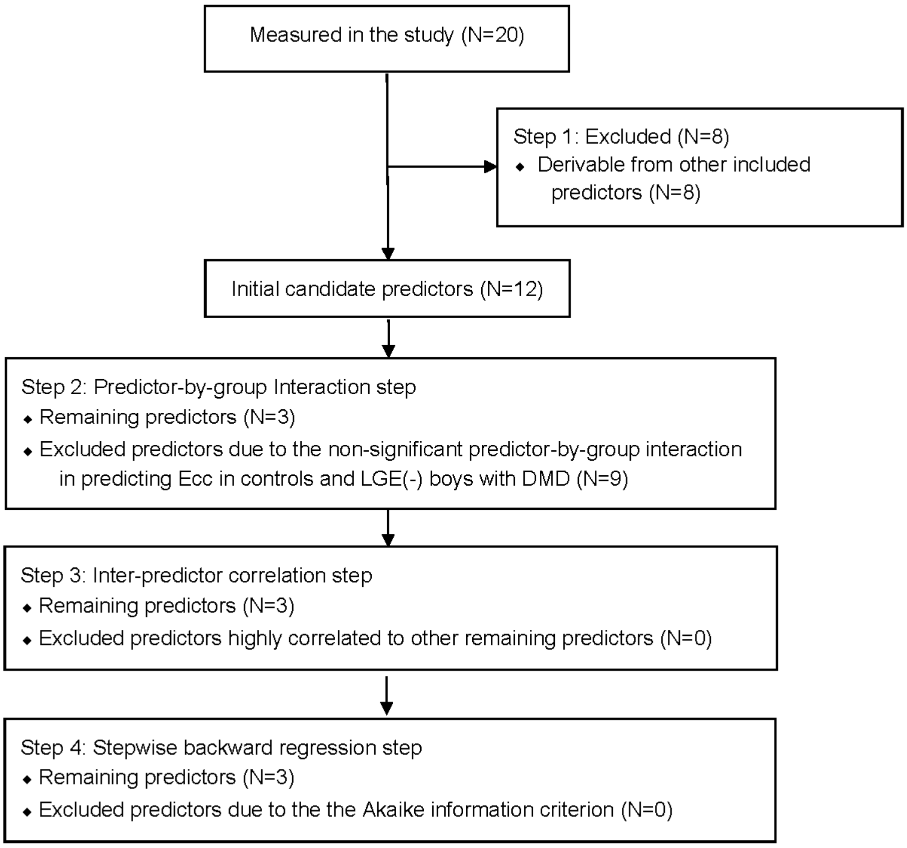

Figure S1. Flow diagram using the CONSORT [39] for building a best fitting regression model that predicts the E_{cc} in healthy controls and DMD boys with LGE(-).

Subsequently, the predictor-by-group interaction for each predictor was tested using a group-wise single-predictor regression model to fit the values of pooled E_{cc} in LGE(-) boys with DMD and healthy controls [40]:

$$\textit{Pooled } E_{cc} \sim constant + group + X_i + group \times X_i \qquad \text{(S1)}$$

where X_i is a predictor of interest and group values were assigned as either 0 for controls or 1 for DMD. X_i was removed from the pool of candidate predictors if its corresponding interaction term $group \times X_i$ was not significant (p-value > 0.05). The remaining predictors all contributed to predicting the value of E_{cc} in both groups, but that the predictive effect of each predictor depends on the group.

In order to further remove the redundant predictors, the correlations among the remaining predictors were examined using simple correlation analysis [40], followed by the exclusion of highly correlated predictors (correlation coefficients $R^2 > 0.7$), leading to N remaining predictors.

Lastly, the predictive effect of each remaining biomarker was examined. An initial regression model was first acquired by fitting N remaining predictors to E_{cc} in the two cohorts and defined as:

$$\text{Pooled } E_{cc} \sim \text{constant} + \sum_{i=1}^{N} X_i + \sum_{i=1}^{N}\sum_{j=1}^{N}(X_i X_j)_{j>i} \tag{S2}$$

followed by the application of stepwise backwards optimization [41,42] to add or remove predictors using the Akaike information criterion [43], resulting in final predictors with a significant regression coefficient (p-value < 0.05).

S2

Predictors which can be derived from other predictors in the study are defined as:

$$LVESV = 1 - LVEF \times LVEDV \tag{S3}$$

where LVESV stands for Left ventricular End Systolic Volume, LVEF stands for Left ventricular Ejection Fraction, and LVEDV stands for Left ventricular End Diastolic Volume.

$$RVESV = 1 - RVEF \times RVEDV \tag{S4}$$

where RVESV stands for Right ventricular End Systolic Volume, RVEF stands for Right ventricular Ejection Fraction, and RVEDV stands for Right ventricular End Diastolic Volume.

$$BMI = \frac{Weight}{Height^{2}} \tag{S5}$$

where BMI stands for Body Mass Index.

$$BSA = \sqrt{\frac{100 \times Height \times Weight}{3600}} \tag{S6}$$

where BSA stands for Body Surface Area.

$$LVMi = LVM \times BSA^{-1} \tag{S7}$$

where LVMi stands for Left ventricular Mass Index and LVM stands for Left ventricular Mass.

$$RVMi = RVM \times BSA^{-1} \tag{S8}$$

where RVMi stands for Right ventricular Mass Index and RVM stands for Right ventricular Mass.

$$LVESVi = LVESV \times BSA^{-1} \tag{S9}$$

where LVESVi stands for Left ventricular End Systolic Volume Index.

$$LVEDVi = LVEDV \times BSA^{-1} \tag{S10}$$

where LVEDVi stands for Left ventricular End Diastolic Volume Index.

$$RVESVi = RVESV \times BSA^{-1} \tag{S11}$$

where RVESVi stands for Right ventricular End Systolic Volume Index.

$$RVEDVi = RVEDV \times BSA^{-1} \tag{S12}$$

where RVEDVi stands for Right ventricular End Diastolic Volume Index.
